# SORLA-Mediated Trafficking of TrkB Enhances the Response of Neurons to BDNF

**DOI:** 10.1371/journal.pone.0072164

**Published:** 2013-08-19

**Authors:** Michael Rohe, Daniela Hartl, Anja Nawarecki Fjorback, Joachim Klose, Thomas E. Willnow

**Affiliations:** 1 Molecular Cardiovascular Research, Max-Delbrueck-Center for Molecular Medicine, Berlin, Germany; 2 Institute for Medical Genetics and Human Genetics, Charité - University Medicine Berlin, Berlin, Germany; 3 Department of Biomedicine, Aarhus University, Aarhus, Denmark; University of Louisville, United States of America

## Abstract

Stimulation of neurons with brain-derived neurotrophic factor (BDNF) results in robust induction of SORLA, an intracellular sorting receptor of the VPS10P domain receptor gene family. However, the relevance of SORLA for BDNF-induced neuronal responses has not previously been investigated. We now demonstrate that SORLA is a sorting factor for the tropomyosin-related kinase receptor B (TrkB) that facilitates trafficking of this BDNF receptor between synaptic plasma membranes, post-synaptic densities, and cell soma, a step critical for neuronal signal transduction. Loss of SORLA expression results in impaired neuritic transport of TrkB and in blunted response to BDNF in primary neurons; and it aggravates neuromotoric deficits caused by low BDNF activity in a mouse model of Huntington’s disease. Thus, our studies revealed a key role for SORLA in mediating BDNF trophic signaling by regulating the intracellular location of TrkB.

## Introduction

Sorting protein-related receptor containing LDLR class A repeats (SORLA) is a member of the VPS10P domain receptor gene family, a class of sorting proteins expressed in the mammalian nervous system [1]. Typically, VPS10P domain receptors shuttle between Golgi, plasma membrane, and early endosome and direct intracellular trafficking of target proteins to distinct subcellular compartments in neurons. SORLA is best recognized for its role as a sorting receptor for the amyloid precursor protein (APP), the main etiologic agent in Alzheimer disease (AD). SORLA interacts with APP to prevent routing of the precursor protein into late endosomes where proteolytic breakdown into neurotoxic amyloid-β peptides (Aβ) occurs [2], [3]. SORLA activity is perceived as neuroprotective as it reduces the extent of Aβ production and senile plaque deposition in mouse models [4], [5] and in patients with sporadic AD [6].

Recently, the relevance of SORLA as a neuroprotective factor received independent support by findings that *Sorl1* (the gene encoding SORLA) is a downstream target of brain-derived neurotrophic factor (BDNF), a growth factor that signals through tropomyosin-related kinase receptor (TrkB) to promote neuronal survival [7], [8]. In neurons, BDNF signaling increases *Sorl1* transcription 10-fold whereas absence of BDNF activity in mouse models with genetic (*Bdnf ^−/−^*) or disease-related loss of BDNF in the brain (Huntington’s disease, HD) results in impaired SORLA expression and activity [9]. However, the question why SORLA is part of the trophic response of neurons to BDNF stimulation remained unanswered.

Using proteomics approaches combined with functional studies in cultured neurons and in mouse models of HD, we now identified SORLA as novel sorting factor for TrkB that facilitates transport of this BDNF receptor along neurites to enhance BDNF signaling. Thus, BDNF-mediated induction of SORLA expression may represent a cellular mechanism to potentiate trophic signals, a pathway potentially disrupted in HD.

## Materials and Methods

### Ethics Statement

All experiments performed with mice were conducted according to the guidelines of the German Animal Welfare Law. The study was approved by the State Office of Health and Social Affairs Berlin (approval number X9012/12).

### Mouse Models, Animal Experimentation and Reagents

Generation of the Huntington’s disease mouse model (B6C3-Tg(HD82Gln)81Dbo/J) carrying a transgenic huntingtin gene with 82 CAG repeats [10] and animals genetically deficient for *Sorl1^−/−^* [2] has been described before. For rotarod performance analysis (adapted from [11]) equal numbers of mice of either genotype matched for age and sex were analyzed on four consecutive days for their latency to fall off the accelerating rotating rod (total n = 8–10; males n = 4–5, females n = 4–5).

Antisera have been obtained from Cell Signaling Technology or Santa Cruz Biotechnology. Plasmids coding for TrkB-mCherry, TrkB-EGFP, and SORLA-EGFP have been generated by introducing corresponding cDNA into the pCI Mammalian Expression Vector (Promega) and pcDNA3 vector (Invitrogen), respectively.

### Preparation and Treatment of Primary Neurons

Primary cortical neurons were prepared from newborn Balb/c mice of either sex at postnatal day 1. Cortices were dissociated in papain (1 hour at 37°C) and cultured on poly-D-lysine/collagen coated culture dishes. The neurons were cultured for 5 days in Neurobasal-A medium (Gibco) including B27 supplement (Sigma), and GlutaMAX (Invitrogen) as previously described [9].

Neurons were treated with BDNF (150 ng/ml, Tebu-Bio) or medium only (control) for 20 minutes (BDNF/TrkB signaling) or 48 hours (BDNF-dependent proteome changes) by replacing half of the culture medium with fresh medium. For proteome analyses the cells were harvested in ice cold PBS and cell pellets were frozen immediately in liquid nitrogen. Six individual samples of each treated and control cells were collected (n = 6).

### Protein Extraction and 2-D Electrophoresis

For proteome analyses, the cells were harvested in ice cold PBS. Six individual samples of each treated and control cells were collected (n = 6). Protein extracts were prepared from frozen cell pellets and separated by large-gel 2D-PAGE technique as described [12], [13], [14]. Protein spot patterns were evaluated using Delta2D imaging software (version 4.0, Decodon). Percent volume of spot pixel intensities was used for quantitative analysis of protein expression by Delta2D as described before [15]. One-way ANOVA including permutation-based false discovery rate correction (FDR ≤10%) was applied to determine statistical significance of alterations (significance threshold p≤0.05; Delta2D). Normal distribution of data was validated before testing using the Kolmogorov-Smirnov test (Prism, GraphPad; version 5.0c). Only fold changes exceeding 20% were considered.

### Mass Spectrometry

For protein identification, 1200 µg protein extract was each separated on a 2-D gel and stained with a MS-compatible silver staining protocol [12]. Protein spots of interest were excised from gels and subjected to in-gel tryptic digestion as described [12]. Peptides were characterized by an ESI-tandem-MS/MS on a LCQ Deca XP ion trap instrument (Thermo Finnigan, Waltham, MA). Mass spectra were evaluated using MASCOT (version 2.1) automatically searching SwissProt database (version 51.8/513877 sequences). MS/MS ion search was performed with the following set of parameters: (i) taxonomy: Mus musculus, (ii) proteolytic enzyme: trypsin, (iii) maximum of accepted missed cleavages: 1, (iv) mass value: monoisotopic, (v) peptide mass tolerance 0.8 Da, (vi) fragment mass tolerance: 0.8 Da, (vii) fixed modifications: none and (viii) variable modifications: oxidation of methionine and acrylamide adducts (propionamide) on cysteine. Only proteins with scores corresponding to p<0.05, with at least two independent peptides identified were considered. The cut-off score for individual peptides was equivalent to p<0.05 for each peptide as calculated by MASCOT.

#### Subcellular fractionation studies

Brain tissue was homogenized using a glass-Teflon Potter S homogenizer with 10 strokes at 900 rpm on ice. Homogenates were cleared through differential centrifugation. Synaptosomes were subjected to osmotic shock and put on a discontinous sucrose gradient (1.2 M, 1.0 M, 0.8 M). Further enrichment of different fractions was achieved through additional steps of centrifugation as described [16], [17].

### Cell Culture Studies

SH-SY5Y cells [18], [19] or primary neurons were transfected with expression plasmids using Lipofectamine 2000 (Invitrogen). Co-immunoprecipitation of endogenous proteins from brain lysates was conducted according to standard protocols utilizing streptavidin agarose beads (Roche). Surface biotinylation was carried out using the Cell Surface Protein Isolation Kit (Pierce). For time-lapse microscopy, primary neurons were transfected the day after seeding with an expression plasmid encoding EGFP-tagged human TrkB. The day after transfection, the cells were imaged on a Zeiss confocal LSM 510 META microscope at 37°C using a 40× NA 1.2 C-Apochromat objective. Movies were generated by obtaining images at 1 frame every 2 s for 300 s per neurite with a 60× oil-immersion objective (NA 1.4). A total of 186 (*Sorl1^+/+^*) and 228 vesicles (*Sorl1^−/−^*) in 13–16 neurons per genotype were analyzed (three independent experiments). Vesicle movement was quantified by generation of kymostacks using ImageJ KymoToolbox plugin (http://rsb.info.nih.gov/ij/; available on request at Fabrice.Cordelieres@curie.fr).

## Results

### Reduced Proteomic Response in SORLA-deficient Neurons Following BDNF Treatment

**Figure 1:**
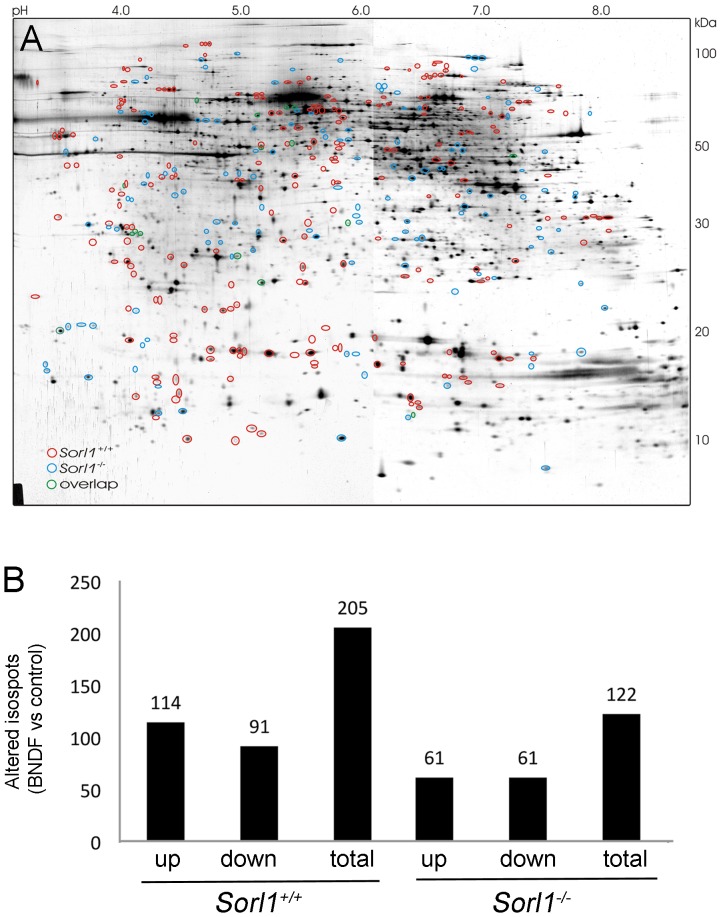
The global BDNF-dependent proteome response is altered in SORLA-deficient neurons. (**A**) A prototypic two-dimensional polyacrylamid gel of proteins from *Sorl1^−/−^* primary cortical neurons stained with silver nitrate. Significant protein spot alterations in response to BDNF treatment (150 ng/ml for 2 days) in wild type neurons (red circles), in SORLA-deficient neurons (blue circles) or in both genotypes (green circles) as compared to untreated samples are indicated. (n = 6 biological replicates per genotype; Student’s *t*-test p<0.05). (**B**) Total number of protein spots altered in primary cortical neurons of the indicated genotypes in response to BNDF treatment as exemplified in panel A. (n = 6 biological replicates per genotype; Student’s *t*-test p<0.05).

Treatment of primary neurons with BDNF results in a strong increase in SORLA mRNA and protein levels [9]. To determine what effect SORLA may have on the trophic response of neurons, we performed two-dimensional polyacrylamide gel electrophoresis (2D-PAGE) comparing the proteomic adaptations of primary neurons either wild type (*Sorl1^+/+^*) or genetically deficient for SORLA (*Sorl1^−/−^*) to BDNF stimulation. Following the experimental conditions of earlier studies on chronic *Sorl1* induction [9], primary cortical neurons of *Sorl1^+/+^* or *Sorl1^−/−^* mice were treated for 48 hours with 150 ng/ml BDNF or medium only (control). The global response (Fig. 1A) and the total number of proteins altered in expression (Fig. 1B) differed markedly between genotypes. Using mass spectrometry, a number of physiological BDNF targets were identified that were differentially regulated in wild type versus SORLA-deficient neurons. These targets included collapsin response mediator protein-2, eukaryotic elongation factor 1A, and stathmin (see Table 1 for details).

**Table 1:** Selected list of proteins differentially regulated in SORLA-deficient versus wild type neurons following chronic BDNF application.

Protein	*Sorl1^−/−^*	*Sorl1^+/+^*	Function
CRMP2	n.a.	↑ 1.96	CRMP2 links TrkB with kinesin-1 to regulate axonal transport of TrkB-containing vesicles [31]. Absent CRMP2 induction in *Sorl1^−/−^* neurons argues for perturbed TrkB trafficking and signaling.
eEF1A	n.a.	↑ 1.42	BDNF increases eucaryote elongation factor 1A (eEF1A) phosphorylation to enhance translation elongation [32]. Reduced eEF1A levels in *Sorl1^−/−^* neurons suggests impaired regulation of synaptic vesicle trafficking.
Stathmin	n.a.	↓ 0.71; 0.73;0.75; 0.78	Chronic application of BDNF reduces phosphorylation of stathmin [33]. Lack of stathmin suppression in *Sorl1^−/−^* neurons suggests reduced BDNF signaling.

The values indicate ratio of protein spot volume changes (increase ↑ or decrease ↓; ANOVA, p<0.05). Where applicable, data for different iso-spots of the same protein are given. n.a., not altered.

### Reduced Signal Cascade Activation in SORLA-deficient Neurons after BDNF Treatment

**Figure 2:**
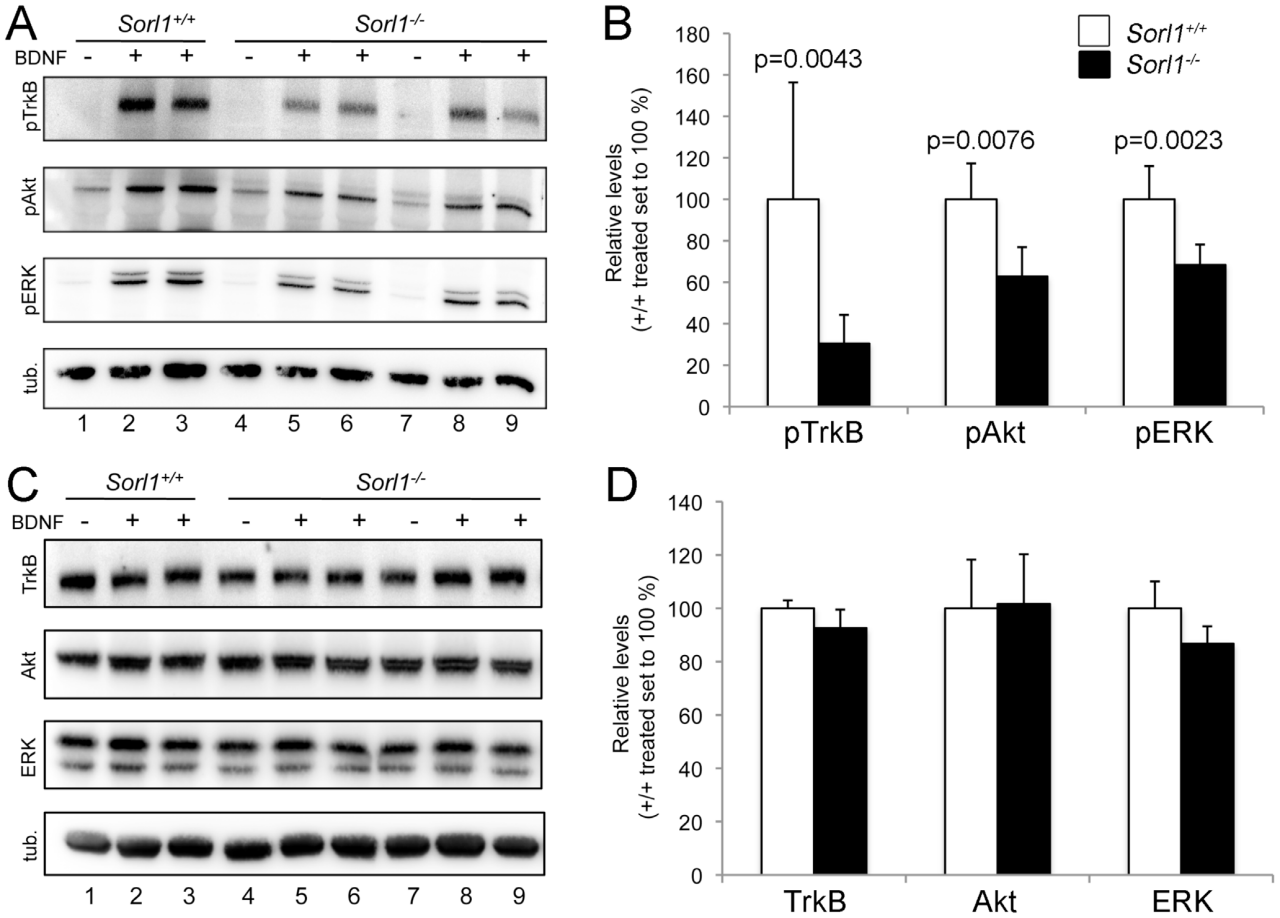
Reduced BDNF-dependent activation of TrkB in SORLA-deficient neurons. (**A, B**) Quantification of phosphorylated (p) forms of TrkB, Akt, and ERK in primary cortical neurons either non-treated (−) or treated with 150 ng/ml BDNF for 20 min (+) using Western blotting (A) and densitometric scanning of replicate blots (B). *Sorl1^−/−^* neurons show reduced levels of pTrkB, pAKT, and pERK compared with *Sorl1^+/+^* cells (n = 6–12, Mann-Whitney U test). (**C, D**) Quantification of total levels of TrkB, Akt, and ERK in primary neurons either non-treated (−) or treated with 150 ng/ml BDNF (+) for 20 min using Western blot (C) and densitometric scanning of replicate blots (D). Detection of tubulin (tub.) served as loading controls in A and C.

In neurons, BDNF binding to TrkB results in phosphorylation-dependent activation of TrkB and in down-stream signaling through the extracellular regulated kinase (ERK) and Akt pathways [7]. Thus, we analyzed levels of posphorylated (active) forms of TrkB (pTrkB), as well as of signal mediators ERK (pERK) and Akt (pAkt) in primary neurons following acute BDNF application (20 min, 150 ng/ml). Western blot analysis revealed significant activation of TrkB, ERK, and Akt in both genotypes after BDNF addition as the phosphorylation of all three proteins was induced upon stimulation. However, comparing the strength of signal induction documented markedly reduced activation of all three signaling cascade components in *Sorl1^−/−^* compared with *Sorl1^+/+^* neurons (Fig. 2A and B). In contrast, the total levels of TrkB, ERK, and Akt did not vary between genotypes (Fig. 2C and D).

Taken together, the analysis of signal cascade activation identified a blunted response to BNDF in neurons lacking SORLA despite proper expression of TrkB.

### SORLA Interacts with TrkB and Determins TrkB Surface Expression

Functional expression of Trk receptors in neurons involves extensive axonal transport processes whereby these receptor molecules shuttle between cell body and synapses (reviewed in [20]). Because SORLA acts as neuronal sorting receptor for APP, we tested whether the protein may have a similar role in intracellular transport of TrkB.

**Figure 3:**
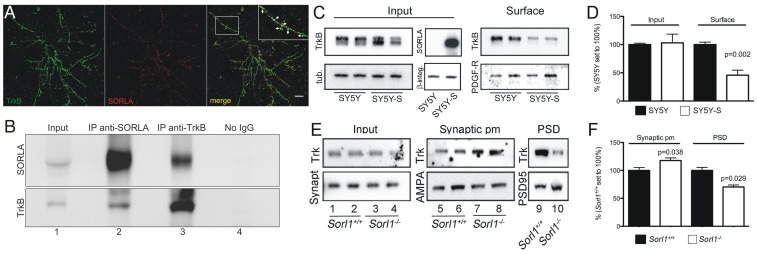
Interaction with SORLA controls trafficking and synaptic exposure of TrkB. (**A**) Colocalization of endogenous SORLA (red) and TrkB (green) in primary cortical neurons as shown by confocal immunofluorescence microscopy. Scale bar: 10 µm. (**B**) Co-immunoprecipitation of endogenous SORLA and TrkB from brain lysates is seen using anti-SORLA (IP anti-SORLA; lane 2) and anti-Trk antisera (IP anti-TrkB; lane 3). Panel Input (lane 1) indicates presence of endogenous SORLA and TrkB in brain lysate prior to co-immunoprecipitation. Lane 4 indicates lack co-immunoprecipitation in the absence of primary antibody (No IgG). (**C**) SH-SY5Y neuroblastoma cells stably overexpressing SORLA (SY5Y-S) or parental control cells (SY5Y) were transfected with expression constructs for TrkB. Subsequently, proteins at the cell surface were biotinylated and immunoprecipitated using streptavidin beads. Western blot analysis documents reduced levels of biotinylated TrkB at the cell surface in SY5Y-S compared to SY5Y cells (panel Surface). Panel Input represents levels of TrkB and SORLA in cell lysates prior to precipitation. Detection of tubulin (tub.), β-integrin (β -integ.), and PDGF-β receptor (PDGF-R) served as controls for loading and immunoprecipitation, respectively. (**D**) Densitometric quantification of replicate Western blots as exemplified in (C) (n = 6, Student’s *t*-test). (**E**) Subcellullar fractionations of wild type and SORLA-deficient mouse brain extracts were probed for the indicated proteins using Western blot analysis. Elevated levels of TrkB receptors (pan-Trk antibody) are seen in the synaptosomal plasma membrane (pm) fraction in SORLA-deficient (lanes 7 and 8) as compared to control brains (lanes 5 and 6). In contrast, levels of Trk receptors in the postsynaptic density (PSD) are reduced in receptor-deficient (lane 10) compared with wild type brains (lane 9). Levels of Trk in total brain lysates prior to subcellular fractionation are similar between genotypes (lanes 1–4). Detection of synaptophysin (Synapt), AMPA receptors, and PSD95 served as respective loading controls. (**F**) Densitometric quantification of replicate Western blots as exemplified in (E) (n = 4–6, Student’s *t*-test).

Using immunofluorescence microscopy, co-localization of endogenous TrkB and SORLA was shown in primary neurons (Fig. 3A). Co-immunoprecipitation of endogenous proteins from brain lysates further documented physical interaction of TrkB with SORLA (Fig. 3B) (lanes 2 and 3).

To explore the relevance of SORLA activity for TrkB trafficking, we tested the subcellular localization of the receptor in the presence or absence of SORLA. Using surface biotinylation experiments, significantly fewer TrkB molecules were detected at the surface of neuroblastoma cells expressing SORLA (SY5Y-S) compared to parental control cells (SY5Y) lacking the protein (Fig. 3C and D). A shift in subcellular localization of TrkB was also seen in the murine brain in response to SORLA expression assubcellular fractionation studies documented increased levels of TrkB in the synaptic plasma membrane fraction and decreased levels in the postsynaptic density fraction of mice lacking SORLA compared with wild-type controls (Fig. 3E and F).

Together, our results demonstrate that SORLA interacts with TrkB to determine TrkB surface expression. Loss of SORLA results in accumulation of TrkB at the cell surface. This is in line with our previous result demonstrating blunted signal cascade activation as this process depends on internalization and transport of activated TrkB receptors to intracellular compartments.

### SORLA Regulates Anterograde and Retrograde TrkB Transport

Altered subcellular localization of TrkB and corresponding changes in TrkB signaling in SORLA-deficient neurons may be caused by defects in either anterograde, retrograde, or bi-directional transport of the BDNF receptor. Accordingly, we applied time-lapse microscopy to measure movement of vesicles containing a fluorescently labeled fusion protein of TrkB with EGFP in *Sorl1^+/+^* and *Sorl1^−/−^* primary neurons.

**Figure 4:**
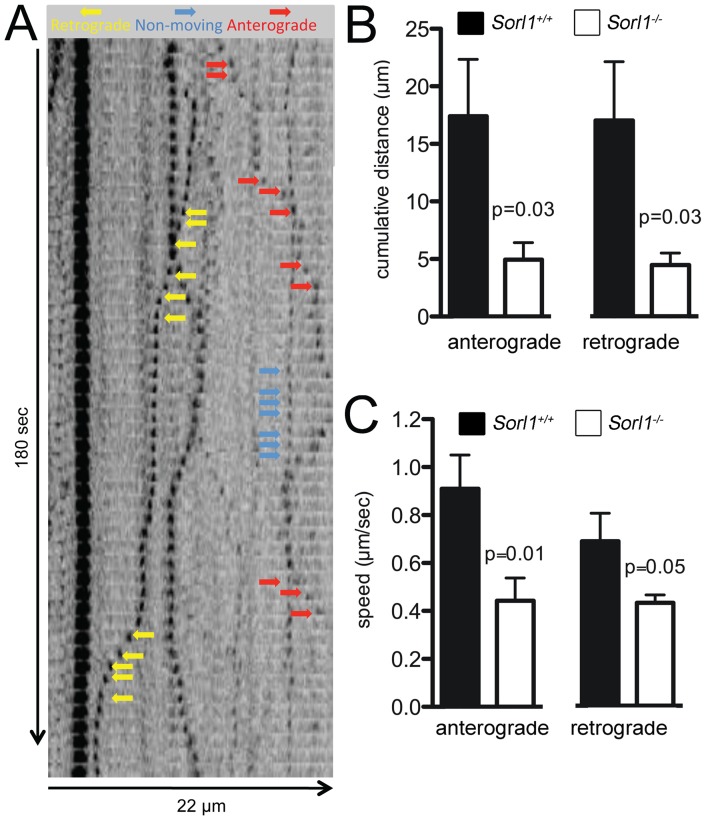
Loss of SORLA impairs vesicular trafficking of TrkB. (**A**) Movement of TrkB-EGFP in neurites of cultured hippocampal neurons. Arrows indicate anterograde (red) and retrograde movement (yellow), or no movement (blue) of vesicles. Images were captured every 2 sec. (**B**) The cumulative distance travelled by TrkB-EGFP fusion proteins either anterogradely or retrogradely is significantly reduced in *Sorl1^−/−^* neurons as compared to controls (n = 14–16, Student’s *t*-test). (**C**) The cumulative anterograde or retrograde speed of the TrkB-EGFP fusion protein is significantly reduced in *Sorl1^−/−^* neurons as compared to *Sorl1^+/+^* cells (n = 14–16, Student’s *t*-test).

As shown in Fig. 4, both anterograde and retrograde trafficking of TrkB-EGFP containing vesicles was impaired in SORLA-deficient neurons with a significant reduction in cumulative distance (Fig. 4B) and speed (Fig. 4C) traveled.

Taken together, analysis of TrkB localization in cells and in the brain as well as evaluation of TrkB trafficking in transfected primary neurons argued for a role of SORLA in transport of TrkB.

### SORLA Deficiency Aggravates Disease Phenotype in a Mouse Model for Huntington's Disease

In HD, levels of BDNF are distinctly reduced in the striatum of affected individuals [21], [22]. In mouse models of the disease (HD82 mice; see method for details of the model) [10] reduced BDNF activity in the striatum coincides with a significant reduction in expression of the BDNF target SORLA in the very same brain area to a similar extent [9]. Based on SORLA’s ability to promote neuritic transport and signaling through TrkB, loss of SORLA expression in HD may potentiate phenotypes caused by low BDNF levels. To test this hypothesis, we generated HD82 mice lacking SORLA (HD82x*Sorl1^−/−^*) and compared them to control HD82 animals (HD82x*Sorl1^+/+^*).

**Figure 5:**
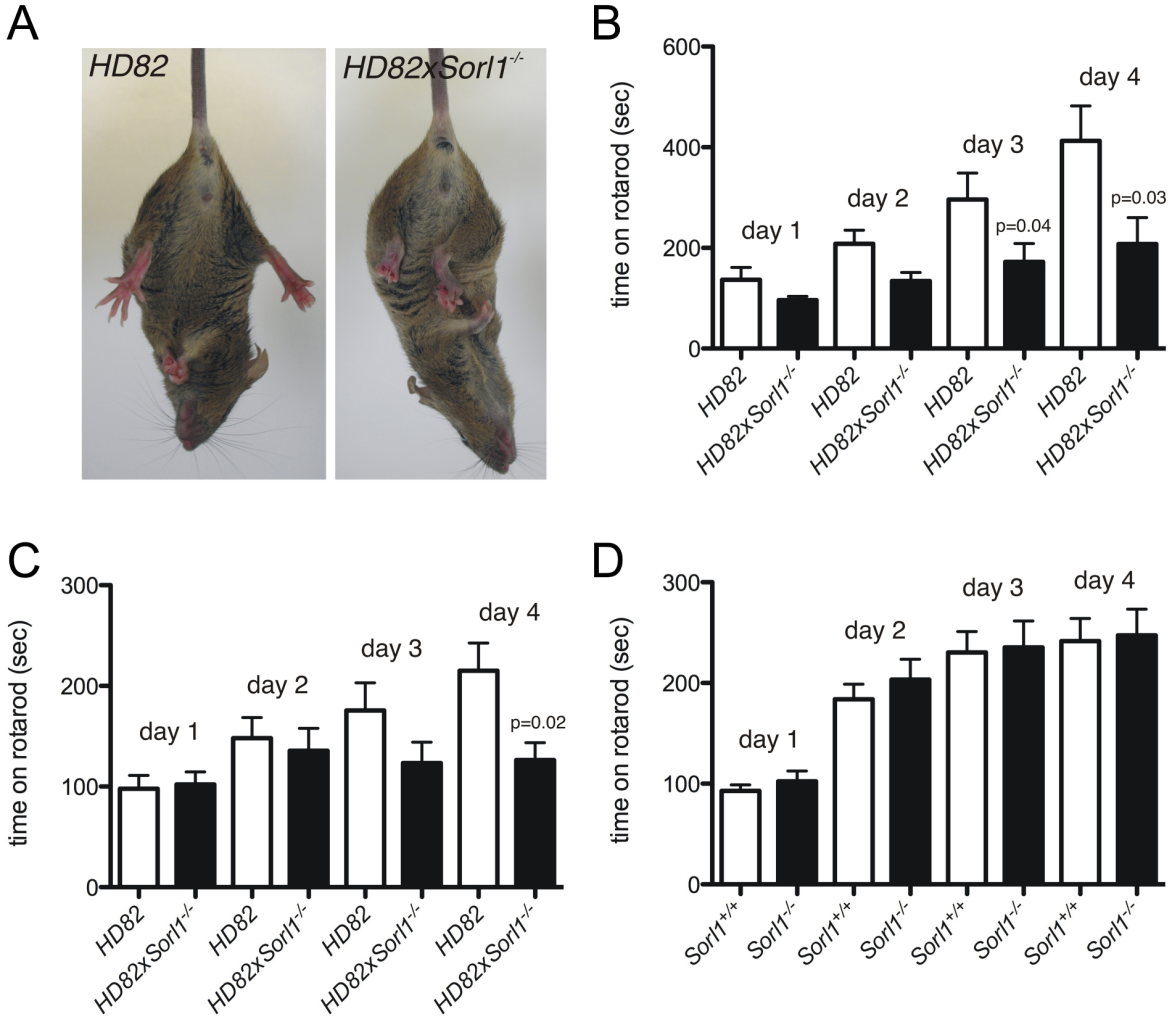
Loss of SORLA aggravates neuromotoric deficits in Huntington’s disease mice. (**A**) At 10 weeks of age, Huntington’s disease mice deficient for SORLA (HD82x*Sorl1^−/−^*) display aggravated hind limb clasping compared with HD82 control littermates (HD82x*Sorl1^+/+^*). (**B, C**) Worsening of neuromotoric deficits due to loss of SORLA is also seen when comparing (HD82x*Sorl1^−/−^*) animals with (HD82, *Sorl1^+/+^*) controls of either sex at 10 (B) and 18 weeks of age (C) during consecutive days of rotarod performance test (n = 8–9, Mann-Whitney-U). (**D**) No difference in rotarod performance is seen comparing 10 weeks-old *Sorl1^+/+^* and *Sorl1^−/−^* mice matched for age and sex (n = 10, Mann-Whitney-U).

At 10 weeks of age, HD82x*Sorl1^−/−^* mice displayed pronounced hind limb clasping compared with HD82 control litter mates (HD82x*Sorl1^+/+^*) when suspended by their tail (Fig. 5A). Abnormal hind limb posture as a consequence of impaired neuromotoric activity has been observed in HD82 mice before. However, this phenotype typically does not appear before 16–22 weeks of age [10]. Worsening of neuromotoric deficits in HD82 mice lacking SORLA was also substantiated by rotarod performance testing at 10 weeks of age. While HD82 mice showed a significant improvement in performance when tested for four consecutive days, HD82x*Sorl1^−/−^* failed to do so and performed significantly poorer on day 3 and 4 (Fig. 5B). This phenotype was also seen at 18 weeks of age (Fig. 5C). Neuromotoric deficits were not observed when comparing *Sorl1^−/−^* with wild-type controls (*Sorl1^+/+^*) (Fig. 5D), indicating that behavioral defects in HD82x*Sorl1^−/−^* mice were caused by aggravation of HD-related phenotypes.

## Discussion

Using screening approaches in primary neurons, we previously identified BDNF as a major inducer of *Sorl1* that activates receptor gene transcription through the ERK pathway. Induction of *Sorl1* transcription results in a robust increase in SORLA protein expression and activity in cultured neurons and in the mouse brain *in vivo* [9]. A clue towards understanding the physiological relevance of SORLA as downstream target of BDNF signaling came with the proteomics analyses in this study that documented a blunted response of SORLA-deficient neurons to BDNF application (Figs. 1 and 2). The underlying molecular mechanism was revealed in studies in cultured neurons demonstrating a hitherto unknown function for SORLA in axonal trafficking of TrkB, a step critical for efficient neurotrophin signal transduction (Figs. 3 and 4).

To elicit its biological effects, BDNF signals must be conveyed over long distances from the nerve terminal to the cell body [23]. In detail, nascent TrkB receptor molecules are targeted anterogradely along the axonal path to the synapse. Following neurotrophin binding to Trk receptors, receptor-ligand complexes internalize in signaling endosomes and signaling persists within these vesicles as they move retrogradely back to the cell body to control gene expression [7], [24]. The detailed mechanisms of TrkB transport are not fully resolved. Conceptually, our studies support a model whereby physical interaction with SORLA facilitates neuritic transport of TrkB to and from the synapse, promoting BDNF signal transduction from nerve termini to cell soma. Lack of SORLA, as in primary *Sorl1^−/−^* neurons, results in enhanced accumulation of TrkB at the synaptic plasma membrane and in depletion from the post-synaptic density compartment (Fig. 3E and F). Also, anterograde as well as retrograde transport of TrkB along neurites is delayed in the absence of the receptor (Fig. 4). Several cytosolic adaptor proteins, including GGA (Golgi-localized, gamma adaptin ear-containing, ARF-binding protein), phosphofurin acidic cluster sorting protein 1, and retromer have been shown to interact with the carboxy-terminal tail of SORLA and to direct its intracellular trafficking path [3], [25]. Potentially, these interactions not only govern SORLA-dependent transport of APP but of TrkB as well. Clearly, the molecular mechanisms controling SORLA-dependent antero/retrograde sorting of TrkB at the synapse and its relevance for promotion of signal transduction still await elucidation.

A similar function as for SORLA in trafficking of TrkB has been documented for sortilin, a related receptor,of the VPS10P domain receptor family [26]. Unlike sortilin, that mainly regulates anterograde transport of TrkB, SORLA apparently controls both, anterograde and retrograde sorting of this receptor. In addition, *Sorl1* but not the gene encoding sortilin is induced by BDNF in neurons [9]. Thus, SORLA appears as integral part of a self-potentiating activation loop whereby BDNF distinctly induces SORLA expression to accelerate axonal transport of its cognate receptor and to strengthen trophic signals. Further support for the specificity of this activation loop stems from the fact that BDNF but not other neurotrophins (such as NGF) induced *Sorl1* [9].

BDNF signaling has long been recognized as an autocrine mechanism whereby adult sensory neurons secrete BDNF to sustain their own survival signals [27]. A more complicated scenario is true in the striatum where BDNF produced in cortical neurons needs to be transported to striatal neurons to support their survival. Striatal neuronal cell loss due to insufficient BDNF support has been recognized as a molecular mechanism in HD, a monogenic disease caused by a polyglutamine expansion (polyQ) in huntingtin. Huntingtin is a protein that facilitates vesicular transport of BDNF along microtubules. Compared to the wild-type protein, the polyQ-mutated huntingtin fails to efficiently transport BDNF, resulting in loss of trophic support and in neuronal toxicity [28]. Experimental evidence suggests that BDNF arrives in the striatum by anterograde transport from the cortex via cortico-striatal afferents [29], [30]. We have reported before that in Huntington diseased mice (HD82) low levels of striatal BDNF result in low levels of SORLA [9]. Now, we show that complete loss of SORLA expression in HD82 mice aggravates the neuromotoric decline in this model of HD (Fig. 5). These findings further support the pathophysiological relevance of impaired BDNF trophic support in HD, and the relevance of SORLA as neuronal sorting receptor for TrkB in the molecular pathogenesis of this disease.
